# An inverse method for determining the spatially resolved properties of viscoelastic–viscoplastic three-dimensional printed materials

**DOI:** 10.1098/rspa.2015.0477

**Published:** 2015-11-08

**Authors:** X. Chen, I. A. Ashcroft, R. D. Wildman, C. J. Tuck

**Affiliations:** Additive Manufacturing and 3D Printing Research Group, Faculty of Engineering, University of Nottingham, NG7 2RD, UK

**Keywords:** nanoindentation test, reactive inkjetting, inverse finite-element analysis, viscoelastic–viscoplastic

## Abstract

A method using experimental nanoindentation and inverse finite-element analysis (FEA) has been developed that enables the spatial variation of material constitutive properties to be accurately determined. The method was used to measure property variation in a three-dimensional printed (3DP) polymeric material. The accuracy of the method is dependent on the applicability of the constitutive model used in the inverse FEA, hence four potential material models: viscoelastic, viscoelastic–viscoplastic, nonlinear viscoelastic and nonlinear viscoelastic–viscoplastic were evaluated, with the latter enabling the best fit to experimental data. Significant changes in material properties were seen in the depth direction of the 3DP sample, which could be linked to the degree of cross-linking within the material, a feature inherent in a UV-cured layer-by-layer construction method. It is proposed that the method is a powerful tool in the analysis of manufacturing processes with potential spatial property variation that will also enable the accurate prediction of final manufactured part performance.

## 1. Introduction

In reactive inkjet printing (RIJ), pico-volume droplets of fluid materials are jetted onto the surface of a substrate, or a previously printed layer surface, and a chemical reaction, or physical interaction, occurs *in situ*, leading to the creation of a new material during the manufacturing process [1]. This method has particular appeal for three-dimensional (3D) printing since the process of repeated deposition and solidification can be used to manufacture 3D components. A range of material types can be produced using RIJ techniques, including polymers [2–4], mixed oxide [5], metals [6], gels [7] and semiconductors [8]. An added benefit of the layer-by-layer approach is that if control over the material creation process can be exerted, then there is significant potential for variation in material properties across the volume of a part. Before being able to achieve such control, however, it is necessary to understand the influence of the manufacturing technique on the properties of the material as a function of both time and position. Traditional characterization techniques, such as tensile, creep and relaxation tests, are not able to resolve spatial variations in properties. One method that has the potential for achieving this is nanoindentation (also known as depth sensing indentation), which is able to quantitatively resolve variation in mechanical properties at the micrometre scale, raising it as a possible technique for assessing variation within RIJ built components [9–12].

One disadvantage of nanoindentation, however, is the difficulty in interpreting the test results [13]. The standard extracted material parameters of reduced modulus and hardness are suitable for characterizing elastic–plastic materials [14], such as metals, but less useful for viscoelastic (VE) materials. Some efforts have been made to generate useful data from the nanoindentation of polymers by removing the effects of time-dependent material behaviour on elastic modulus measurements by increasing the load-holding stage time [13,15] or by high-order fitting to indentation curves [16]; however, this is not useful in characterizing the important VE behaviour seen in polymers. To overcome this, Oyen [17] proposed analytical techniques to evaluate VE parameters from nanoindentation test displacement curves based on linear VE material models and hereditary integral analysis of polymer indentation data. This was originally limited to linear VE materials and then extended into materials with different constitutive models [18–22].

An alternative, and more flexible, method of extracting time-dependent material parameters from nanoindentation data is to use an inverse finite-element analysis method (IFEM) that deploys a combination of forward model and experimental observation to determine the materials properties through the application of iterative optimization methods [23,24]. Principally, IFEM is similar to the analytical method proposed by Oyen [17] in which hereditary integral analysis was used for the computation of the VE equations and fitted with indentation test reference data. In applying IFEM to nanoindentation, finite-element analysis (FEA) replaces the analytical integration, and optimal parameters are obtained by fitting output from the computational model to indentation reference curves by means of nonlinear minimization techniques. As numerical computation is used in IFEM, complex or nonlinear material constitutive models can be implemented, thus expanding its applicability beyond that of the analytical method. Originally proposed for geological problems, where direct determination of ground properties was not possible [25–27], IFEM has been shown to be an effective tool for the *in situ* estimation of rate-dependent material properties from non-standard tests [28,29].

IFEM has been used to extract material parameters for various materials when combined with nanoindentation tests. Erdemir *et al*. [30] reported the development of a numerical–experimental approach to characterize heel-pad deformation at the material level. An inverse FEA of the indentation protocol using an axisymmetric model adjusted to reflect individual heel thickness was used to extract nonlinear material properties enabling the hyperelastic behaviour of the heel to be modelled. Sangpradit *et al*. [31] also used a hyperelastic material constitutive relation in their IFEM, combining it with a rolling indentation test to characterize the mechanical properties of a tissue phantom. Hamzah *et al*. [32] applied IFEM to tensile test data, using an anisotropic hyperelastic material model to characterize the fibre orientation in intercostal muscles, and Abyaneh *et al*. [33] developed a hybrid IFEM approach, employing a gradient-based nonlinear minimization algorithm, a VE material constitutive model and nanoindentation test data to extract the anisotropic material parameters of a cornea.

From the literature [28–33], it can be seen that a key step in the use of IFEM is the selection of an appropriate material constitutive model. Only a material constitutive model that can accurately describe the mechanical behaviour of the material is suitable; otherwise, it is impossible to achieve a good fit between FEA and test data. Although linear VE constitutive relations have been used to describe the mechanical behaviour of polymer materials under indentation, tests and analysis are mostly limited to the loading and holding (or creep) stages [34–36] of an indentation test, without considering the unloading stage. It was found in this investigation that not all the linear VE parameters extracted from the loading and holding stages of a test fit well with the unloading stage. The predicted recovery during the unloading stage tends to be much higher than the tested recovery when using the material parameters determined from loading and holding stages. This indicates that there could be viscoplastic (VP) deformation during loading and holding stages besides the viscous and elastic elements. Therefore, a more advanced material constitutive model should be considered if material responses under complex loading scenarios that include unloading are to be accurately modelled.

In this paper, nonlinear viscoelastic (NVE), viscoelastic–viscoplastic (VEVP) and nonlinear viscoelastic–viscoplastic (NVEVP) material constitutive models are developed and compared with the more standard linear VE material behaviour. An IFEM is then developed and used to investigate which material model most accurately describes the mechanical behaviour of an RIJ polymer. The unloading stage is included in the IFEM as this is necessary to ensure the resultant material model and parameters adequately model the polymer under loading conditions seen in service, which would be expected to include loading, constant load and unloading at various times. The proposed method is then tested for its efficacy in determining material model parameters under conditions of 3D RIJ printing. Using a commercial 3D printing system, samples were manufactured and tested with nanoindentation. The IFEM was then used to determine the most appropriate material model, the optimal material parameters and the spatial variation of these parameters in the sample. The aims of the paper, then, are to introduce an IFEM which can be used to determine complex material model parameters from indentation data and to illustrate how these can be used to investigate property variation in a 3D printed (3DP) part, thus providing insight into the manufacturing process and enabling prediction of the mechanical performance of the printed part.

## 2. Methodology

The proposed method for accurately determining the spatial variation of complex material properties requires a method of experimentally determining spatially resolved mechanical properties, constitutive models that accurately represent the behaviour of the material under realistic loading conditions and a method of determining the best-fit material model parameters. In the sections below, the experimental methods are described first, followed by the IFEM used to determine the best-fit model parameters, and finally a number of constitutive models identified as potential candidates for characterizing the polymeric material used in the experimental work are developed.

### (a) Sample preparation

**Figure 1.**
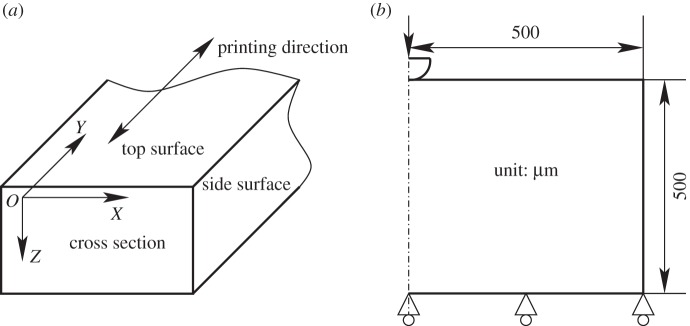
(*a*) Reference coordinate system used in the paper. (*b*) Axisymmetric finite-element domain created to simulate the indentation process.

Samples were prepared using an Object260 Connex 3-D Printer system (Stratasys Ltd). The ink used was VeroClear Fullcure 810, which is a transparent acrylic polymer that is cured after deposition through exposure to UV light. The samples produced were 10×40×5 mm solid blocks (*x*×*y*×*z*; figure 1*a*). These samples were removed from the build plate and sectioned using a hand saw to a size of around 10×5×5 mm. The sectioned samples were then mounted in EpoFix cold-setting resin (Struers Ltd) for nanoindentation. The samples were mechanically machined to create a flat surface, and ground to make the two surfaces parallel. The sample test surface was then polished, finishing with a 1 μm polish. All samples were wrapped with tissue and aluminium foil, to prevent UV exposure, and placed in a desiccator cabinet located in an air-conditioned room in order to minimize any environmental effects prior to testing.

### (b) Nanoindentation

Indentation tests were conducted on a NanoTest 600 machine (Micro Materials Ltd). The maximum loads applied during testing ranged from 25 to 75 mN, with a hold time at maximum load of 500 s. Both the loading and unloading times were fixed at 50 s, meaning that the loading and unloading rates were dependent upon the maximum load applied. The spherical diamond indenter used was 50 μm in radius. All tests were conducted in an enclosed chamber held at 25°C.

The coordinate system used in this paper is shown in figure 1*a*. Surfaces are identified with the following terms: the top surface is the final printed layer of material, creating an *xy*-plane at *z*=0. The side surfaces are the outermost surfaces in the *yz*-plane, i.e. *yz*-planes at the minimum and maximum values of *x*. These are natural surfaces that are formed during the manufacturing process. The normal to this surface is also normal to the print direction. Cross sections were formed by mechanical sectioning in the *xz*-plane. In all the samples, the cross section is by definition at *y*=0. The normal to this surface is parallel to the print direction. Printing was performed in a rastering motion in the print direction.

### (c) Forward finite-element model

The following assumptions were used to establish a working FE model for use in the inverse analysis.

(1) The material is locally homogeneous, i.e. any changes in the material properties are small within a length scale that is longer than the characteristic length scale in the problem, which in this case can be considered to be the contact radius in nanoindentation. Experimental investigation of the rate of change of properties as a function of spatial distance indicated this to be a reasonable assumption for the size of indents used.
(2) The extent of the sample can be considered infinite. In this case, the sample size is two orders of magnitude greater than the indenter size, suggesting that this is appropriate (confirmed by computational investigation of boundary conditions).
(3) The material under indentation can also be considered to be *locally* isotropic, enabling the model to be simplified through axisymmetry.

**Figure 2.**
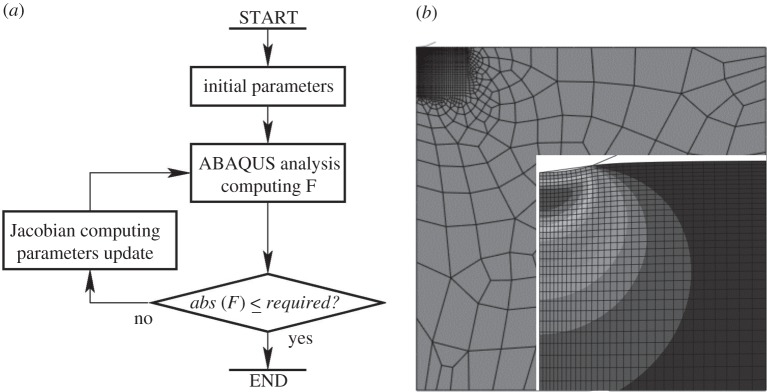
(*a*) Flow chart of the inverse FEA algorithm and (*b*) the axisymmetric FE model mesh used in the inverse analysis, showing the fine mesh deployed around the indenter and the von Mises stress distribution below the indenter for the VE material model.

An axisymmetrical FE indentation model with a 50 μm analytical spherical indenter was, hence, set up, with boundary conditions as shown in figure 1*b*. The representative sample geometry was a 500×500 μm (radius × height) cylinder with indentation performed in alignment with the axis of the cylinder. Tests confirmed that this geometry was sufficient for boundary insensitive results. The mechanical contact between the indenter and the sample material was assumed to be frictionless, tests indicating results to be relatively insensitive to friction at low values. The mesh is shown in figure 2*b* and it can be seen that a fine mesh was applied in the indentation location. Mesh convergence tests were conducted to determine appropriate mesh refinement in the contact area.

### (d) Inverse finite-element modelling technique

Although nanoindentation tests have many advantages over traditional mechanical tests, the evaluated material properties, such as hardness and reduced modulus, are dependent upon the experimental set-up [10] and are not necessarily indicative of intrinsic properties. In addition, the reduced modulus, which is generally determined from the initial region of the load versus depth curve during unloading, may be affected by creep. Inverse FEA modelling, however, is able to accommodate such phenomena, providing the constitutive model employed is sufficient to capture the actual time- and stress-dependent mechanical response of the material.

The IFEM is predicated on the basis that a single combination of material properties and boundary conditions will result in the observed experimental conditions. Proceeding from this assumption, it is possible to iterate the possible material parameters for the given boundary conditions and the given constitutive relation until the properties that lead to results closest to the experimental observations are obtained. IFEM, therefore, provides the possibility of combining the output of the nanoindentation test with an FE model to inversely obtain the material properties, despite the complex stress state in the material induced by the indenter [15]. In the modelling process, a key step is choosing a material constitutive model that is able to accurately represent the mechanical behaviour of the material. This is essential for the method to establish meaningful material property parameters and a good fit between the FEA modelling and the test. In the proposed method, a least-squares objective function is used, given by

2.1 $$\min:F=\sqrt{\frac{1}{n}\Sigma_{i}{\left[S_{t}(t_{i})-S_{F}(t_{i})\right]}^{2}},$$

where *S*_*t*_ is the measured deformation depth at the *i*th time interval, *t*_*i*_; *S*_*F*_ is the depth predicted by the FEA at time *t*_*i*_; and *n* is the total number of time intervals. To match the results from the FEA and experimental tests, linear interpolation was used during the minimization process of equation (2.1) to ensure that the time intervals for the FEA and the test data coincided. In this work, Matlab R2013a was used in combination with ABAQUS 6.11–3 in a gradient-based nonlinear least-squares method for minimization of the objective function. When the relative change of *F* (equation (2.1)) or the change of each searched material property parameters was less than 10^−8^, the minimization process was considered to have converged. Figure 2*a* shows the flow chart for the minimization process together with the mesh used in the axisymmetric FE model (figure 2*b*).

## 3. Material models

In this work, four material constitutive models were evaluated: (i) a linear VE Prony series model, (ii) a VEVP model, (iii) an NVE model, and (iv) an NVEVP model. For the VE model, the intrinsic ABAQUS linear Prony series method [37] was employed. For the VEVP model, the Prony series was combined with a VP model [38]. For the NVE model, Schapery’s equation [39] was combined with a linear Prony series and for the NVEP model this was combined with a VP model. These models are described in the following subsections.

### (a) Linear viscoelastic material model

**Figure 3.**
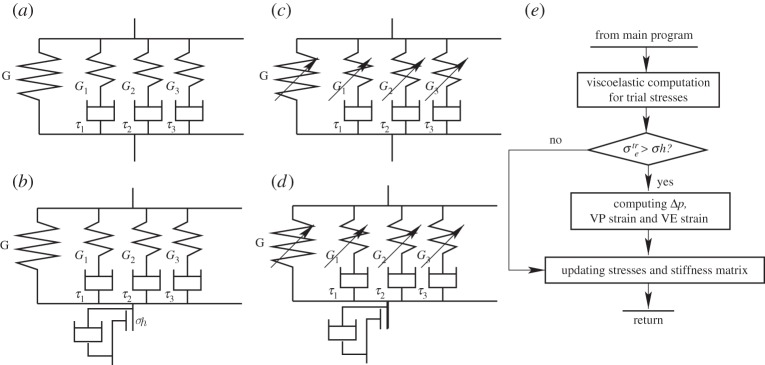
(*a*) VE Prony series material constitutive model, (*b*) VEVP model, (*c*) NVE model, (*d*) NVEVP constitutive model and (*e*) the flow chart of a user-defined material subroutine for the VEVP material model, used in ABAQUS.

The behaviour of many types of polymer can be described by a Prony series model [37,39], which can be represented by springs and dashpots, as shown in figure 3*a*. The instantaneous shear modulus is *G*_0_, the equilibrium modulus is $G_{\infty }$ and the time-dependent modulus *G*(*t*) is given by

3.1 $$G(t)=G_{0}\left\{1-\Sigma_{k}g_{k}\left[1-\exp\left(-\frac{t}{\tau_{k}}\right)\right]\right\},$$

where *g*_*k*_=*G*_*k*_/*G*_0_ and Σ*g*_*k*_<1. In this research, we take the maximum of *k* to be three as additional terms yielded no further improvement in fitting to experimental data. *g*_*k*_ is the relative relaxation modulus and *τ*_*k*_ is the corresponding relaxation time. The linear relationship between stress and strain for an isotropic VE material can be written as

3.2 $$\sigma^{\prime }=2\int_{0}^{t}\left[G_{\infty }+\Sigma_{k}G_{k}\exp\left(-\frac{t-\tau }{\tau_{k}}\right)\right]\frac{d\epsilon^{\prime }}{d\tau }d\tau ,$$

where *σ*′ and *ϵ*′ are the deviatoric stress and strain tensors, respectively.

### (b) Viscoelastic–viscoplastic material model

The VEVP model used in this work is shown in figure 3*b*, i.e. a VE model plus a VP component in series. When the VE von Mises stress in the material is lower than the current yield stress of the material, *σ*_*h*_ (the initial value of *σ*_*h*_ is *σ*_*s*_), of the VP part, the VP component in figure 3*b* is rigid; i.e. there is no VP strain contribution to the total strain and the material behaves as a VE material. In this case, using equation (3.1), equation (3.2) can be rewritten as

3.3 $$\sigma^{\prime }=2G_{0}\left\{\epsilon^{\prime }-\Sigma_{k}g_{k}\left[\epsilon^{\prime }-\int_{0}^{t}\exp\left(-\frac{t-\tau }{\tau_{k}}\right)\frac{d\epsilon^{\prime }}{d\tau }d\tau \right]\right\}.$$

The integration term in (3.3) is the linear Maxwell strain, written as

3.4 $$\epsilon_{k}^{n^{\prime }}=\epsilon^{\prime }-\int_{0}^{t_{n}}\exp\left(-\frac{t_{n}-\tau }{\tau_{k}}\right)\frac{d\epsilon^{\prime }}{d\tau }d\tau .$$

The above equation shows that the Maxwell strain is dependent on time and strain rate. Using (3.4), (3.3) can be written as

3.5 $$\sigma^{\prime }=2G_{0}\left(\epsilon^{\prime }-\Sigma_{k}g_{k}\epsilon_{k}^{n^{\prime }}\right).$$

The above equation shows the linear relationship between strain and stress for a Prony-type VE material, i.e. without VP flow actuated. We refer to the subsequent strain,

3.6 $$\epsilon_{E}^{V}=\epsilon^{\prime }-\Sigma_{k}g_{k}\epsilon_{k}^{n^{\prime }},$$

as the linear VE strain. It can be seen that there is a linear relationship between $\epsilon_{E}^{V}$ and *ϵ*′ from equations (3.6) and (3.4). As the linear VE strain is a function of the Maxwell strain, the linear VE strain is also both time and strain rate dependent. The deviatoric stress, from equations (3.3)–(3.6), can be written as

3.7 $$\sigma^{\prime }=2G_{0}\epsilon_{E}^{V}.$$

The increment format of equation (3.4) has been shown to be [37]:

3.8 $$\Delta \epsilon_{k}^{\prime }=\frac{\tau_{k}}{\Delta t}\left[\frac{\Delta t}{\tau_{k}}+\exp\left(-\frac{\Delta t}{\tau_{k}}\right)-1\right]\Delta \epsilon^{\prime }+\left[1-\exp\left(-\frac{\Delta t}{\tau_{k}}\right)\right](\epsilon^{\prime }-\epsilon_{k}^{n}),$$

and from (3.6)

3.9 $$\Delta \epsilon_{E}^{V}=\Delta \epsilon^{\prime }-\Sigma_{k}g_{k}\Delta \epsilon_{k}^{\prime },$$

where Δ*ϵ*′_*k*_ is the increment of the linear Maxwell strain defined by (3.4) [37]. Equations (3.8) and (3.9) provide a linear VE computation algorithm and define a nonlinear relationship between deviatoric strain increment Δ*ϵ*′ and VE strain increment $\Delta \epsilon_{E}^{V}$ for a given Maxwell strain and time increment, which can be represented by a function *Ω* for later convenience, i.e.

3.10 $$\Delta \epsilon_{E}^{V}=\Omega (\Delta \epsilon^{\prime }).$$

Using (3.7), the deviatoric stress increment is

3.11 $$\Delta \sigma^{\prime }=2G_{0}\Delta \epsilon_{E}^{V},$$

and $\Delta \epsilon_{E}^{V}$ is a time-dependent summable quantity, i.e. $\epsilon_{E}^{V}=\Sigma \Delta \epsilon_{E}^{V}.$ Thus, equations (3.8)–(3.11) can be applied for linear VE computation. Computations have shown that analysis results with these equations implemented within an ABAQUS material subroutine are the same as those with the intrinsic Prony series model in ABAQUS. This provides confidence in extending the approach to viscoplasticity, as described below.

When the VE von Mises stress is higher than the current yield stress *σ*_*h*_ at time *t*, the material is in an ‘overstress’ state [38]; VP flow occurs and a VP flow rate, $\dot{p}$, is actuated:

3.12 $$\dot{p}=\phi (\sigma_{e},\sigma_{h}),$$

where *σ*_*e*_ is the von Mises stress of the material with VE, *σ*_*h*_ is the current yield stress of the material and *ϕ* is a viscous flow function. If, at a time *t*+Δ*t*, a VP deformation occurs due to the actuated $\dot{p}$, an equivalent VP strain increment Δ*p* can be given by

3.13 $$\Delta p=\dot{p}\Delta t=\phi (\sigma_{e},\sigma_{h})\Delta t.$$

For a von Mises-type material, equation (3.13) can be simplified to

3.14 $$\Delta p=\dot{p}\Delta t=\phi (\sigma_{e}-\sigma_{h})\Delta t$$

and can be written in the form

3.15 $$\sigma_{e}=\sigma_{h}+\phi^{-1}(\dot{p}),$$

where *ϕ*^−1^ is the inverse of flow function *ϕ*. Equation (3.15) illustrates the basic difference between elastoplasticity and VP. In elastoplasticity, yielding occurs on the yield surface, and stress is plastic strain rate independent, therefore $\phi^{-1}(\dot{p})\equiv 0$ and *σ*_*e*_≡*σ*_*h*_. In VP, stress is VP strain rate, $\dot{p}$, dependent, thus *σ*_*e*_>*σ*_*h*_, with $\phi^{-1}(\dot{p})>0$. This means that yielding occurs out of the yielding surface for VP. This phenomenon is referred to as ‘over stress’ [38].

One of the flow functions can be assumed to be [38]

3.16 $$\phi =\zeta \;sinh[\eta (\sigma_{e}-r-\sigma_{s})],$$

where *ζ* and *η* are empirical material constants and *σ*_*h*_=*r*+*σ*_*s*_, *σ*_*s*_ is the initial yielding stress and *r* is the monotonically increasing hardening part of the current yielding stress. Combining (3.15) and (3.16) gives

3.17 $$\sigma_{e}=\sigma_{h}+\frac{1}{\eta }\;sinh^{-1}\left(\frac{\dot{p}}{\zeta }\right),$$

where the first term on the right-hand side of (3.17) is the current yield stress and the second part shows the stress due to VP strain rate sensitivity. When a hyperbolic function is used as the flow function, as shown in (3.16), the parameters *ζ* and *η* characterize the amplitude of the stress due to the VP strain rate sensitivity, as seen in (3.17). *η* has the inverse dimensions of stress; while the dimension of *ζ* is the same as strain rate. *ζ* and *η* were selected as variables in the IFEM minimization procedure.

For isotropic hardening, in the case of VEVP behaviour, the yielding stress was assumed to obey a power hardening rule, i.e.

3.18 $$\sigma_{h}=\sigma_{s}{\left[1+A(p+\Delta p)\right]}^{m},$$

where *p* is the total equivalent plastic strain at time *t*. *A* is an empirical material constant, which we assume is equal to 1. Through experiment it was found that a value of *m*=3 led to good fitting for the investigated material. Thus, a constant value of *m*=3 was used to avoid an extra variable in the minimization procedure. Strictly, *m* may be time, strain and strain rate dependent in the VEVP case, especially when strain rate is high; however, in the indentation experiments described in §2b, the strain rate was computed to be low (10^−2^ to 10^−3^). Therefore, the material index parameter is assumed to be strain dependent but strain rate independent in order to simplify parameter fitting in the IFEM. The initial yield stress, *σ*_*s*_, can be considered an intrinsic material parameter and it is expected that this would depend upon the extent of cross-linking in the printed material and therefore may be *z*-dependent.

In the following analysis, an algorithm is developed to calculate the VP yielding the strain tensor Δ*ϵ*_*p*_ increment and corresponding *σ*′ at *t*+Δ*t* when *σ*_*e*_>*σ*_*h*_ for an isotropic VEVP material with isotropic hardening for a given stress tensor ***σ***′_*t*_ at time *t* and the given deviatoric strain increment Δ*ϵ*′ at time *t*+Δ*t*. At time increment *t*+Δ*t* after yield the deviatoric strain increment Δ*ϵ*′ can be divided into two parts, a VE strain increment $\Delta \epsilon_{E}^{V}$ and a VP strain increment Δ*ϵ*_*p*_. From (3.10), the VE strain increment after yielding can be written as

3.19 $$\Delta \epsilon_{E}^{V}=\Omega (\Delta \epsilon^{\prime }-\Delta \epsilon_{p})$$

or

3.20 $${\epsilon_{E}^{V}|}_{t+\Delta t}={\epsilon_{E}^{V}|}_{t}+\Omega (\Delta \epsilon^{\prime }-\Delta \epsilon_{p})$$

and

3.21 $$\sigma^{\prime }{|}_{t+\Delta t}=2G_{0}\epsilon_{E}^{V}{|}_{t+\Delta t}.$$

For VP, it has been pointed out that the normality hypothesis still holds [38]; however, the yield point may occur out of the yield surface, as shown by (3.15) or (3.17). Thus, for a von Mises material we still have

3.22 $$\Delta \epsilon_{p}=\frac{3}{2}\Delta p\frac{\sigma^{\prime }}{\sigma_{e}},$$

where Δ*p* is the equivalent VP strain increment during Δ*t*, as shown by (3.13). Theoretically, a combination of equations (3.13), (3.18), (3.21) and (3.22) forms an implicit nonlinear equation with the unknown Δ*p*. From (3.19), (3.8)–(3.10), we have

3.23 $$\Delta \epsilon_{E}^{V}=\Omega (\Delta \epsilon^{\prime }-\Delta \epsilon_{p})=\Omega (\Delta \epsilon^{\prime })-\Delta \epsilon_{p}\left\{1-\Sigma_{k}g_{k}\frac{\tau_{k}}{\Delta t}\left[\frac{\Delta t}{\tau_{k}}+\exp\left(-\frac{\Delta t}{\tau_{k}}\right)-1\right]\right\},$$

which can be further rewritten as

3.24 $$\Delta \epsilon_{E}^{V}=\Omega (\Delta \epsilon^{\prime }-\Delta \epsilon_{p})=\Omega (\Delta \epsilon^{\prime })-\Delta \epsilon_{p}\hat{1},$$

where

3.25 $$\hat{1}=1-\Sigma_{k}g_{k}\frac{t_{k}}{\Delta t}\left[\frac{\Delta t}{\tau_{k}}+\exp\left(-\frac{\Delta t}{\tau_{k}}\right)-1\right].$$

It can be shown that $\hat{1}$ is a number close to one, dependent on the time increment Δ*t*. Multiplying by 2*G*_0_ on both sides of (3.24) and considering (3.22) and (3.21), we can derive

3.26 $$\sigma^{\prime }\left(1+\frac{3G_{0}\Delta p}{\sigma_{e}}\hat{1}\right)=\sigma^{{\mathrm{tr}}^{\prime }},$$

where

3.27 $$\sigma^{{\mathrm{tr}}^{\prime }}=\sigma^{{\mathrm{tr}}^{\prime }}{|}_{t+\Delta t}=2G_{0}\left[\epsilon_{E}^{V}{|}_{t}+\Omega (\Delta \epsilon^{\prime })\right]+\sigma_{t}^{\prime }+\Delta \sigma^{{\mathrm{tr}}^{\prime }},$$

and

3.28 $$\Delta \sigma^{tr^{\prime }}=2G_{0}\Omega (\Delta \epsilon^{\prime }),$$

where *σ*^tr′^ is the computed VE stress when VP strain Δ*p* is ignored in (3.24), which can be referred to as a trial deviatoric stress tensor. Taking the contracted product of (3.26), we have

3.29 $$\sigma_{e}+3G_{0}\Delta p\hat{1}=\sigma_{e}^{\mathrm{tr}},$$

where $\sigma_{e}^{\mathrm{tr}}$ is the equivalent stress of *σ*^tr′^. Combining (3.26) and (3.29) gives

3.30 $$\text{n}=\frac{3}{2}\frac{\sigma^{\prime }}{\sigma_{e}}\equiv \frac{3}{2}\frac{\sigma^{{\mathrm{tr}}^{\prime }}}{\sigma_{e}^{{\mathrm{tr}}^{\prime }}},$$

where ***n*** is the direction of the VP strain tensor increment Δ*ϵ*_*p*_, as shown by (3.22). The significance of (3.30) is that the direction of the unknown VP strain increment Δ*ϵ*_*p*_ tensor can be determined before Δ*p* is obtained; it is uniquely determined by the current yield surface, regardless of equivalent VP strain Δ*p*. Equations (3.24)–(3.30) are similar to the case of elastoplasticity [38]. Now it is possible to obtain a solution for Δ*p* by combining (3.13), (3.18), (3.29) and (3.30) to form an algebraic nonlinear equation for a von Mises-type material:

3.31 $$\Delta p-\phi (\sigma_{e}^{\mathrm{tr}}-3G_{0}\Delta p\hat{1}-\sigma_{h})\Delta t=0,$$

where $\sigma_{e}^{\mathrm{tr}}$ is computed from equation (3.27) and *σ*_*h*_ can be obtained from the given hardening rule or (3.18), as in this case the direction of VP strain increment has been determined by (3.30), and the trial stress tensor *σ*^tr′^ and $\sigma_{e}^{\mathrm{tr}}$ are already known for the given strain increment Δ*ϵ*′ from (3.27). As both *σ*_*e*_ and *σ*_*h*_ are functions of Δ*p* only, from (3.29) and (3.18), (3.31) is a nonlinear equation involving a scalar unknown, Δ*p*, only. Newton iteration can thus be applied to obtain Δ*p*. The computed Δ*p* can then be applied to (3.24) to update VE strain increment $\Delta \epsilon_{E}^{V},$ and further by (3.11) to update the actual deviatoric stress increment Δ*σ*′. If the computed equivalent trial stress $\sigma_{e}^{\mathrm{tr}}$ is lower than the current yield stress *σ*_*h*_, no VP flow is actuated; thus Δ*p*=0—therefore, from (3.29), the trial equivalent stress $\sigma_{e}^{\mathrm{tr}}$ is the actual equivalent stress *σ*_*e*_ and the corresponding trial stress increment $\Delta \sigma_{e}^{{tr}^{\prime }}$ is the actual deviatoric stress increment.

Comparing equation (3.31) with the elasto-viscoplasticity result in [38], there are two basic differences: (i) $\sigma_{e}^{\mathrm{tr}}$ should be computed using the VE algorithm; (ii) a Δ*t*-dependent number $\hat{1}$ close to one, as shown by (3.25), is included in the equation. These two differences make the current VP flow time, strain rate and VE deformation dependent. This is the reason that this constitutive relation is referred to as a VEVP constitutive material model.

The next step in this work was to write the above algorithm into a material subroutine to enable incorporation in a FEA. The current stress tensor *σ*, strain tensor *ϵ*, strain increment Δ*ϵ* and time increment Δ*t* can be provided by the FE main program; one of the main tasks of the material subroutine is to use the proposed algorithm to compute the corresponding stress increment Δ*σ* based on the information provided by the main code. In order to use the proposed VEVP material model in an FEA in a material subroutine, the stiffness matrix needs to be computed. This will be determined in the next section.

### (c) Schapery’s nonlinear material constitutive model (nonlinear viscoelastic)

For polymers that are intrinsically nonlinear, an NVE constitutive relation may be more appropriate. A linear Prony series from (3.2) can be written as

3.32 $$\sigma^{\prime }=2\left[G_{\infty }\epsilon^{\prime }+\Sigma_{k}G_{k}\int_{0}^{t}\exp\left(-\frac{t-\tau }{\tau_{k}}\right)\frac{d\epsilon^{\prime }}{d\tau }d\tau \right].$$

It is possible to introduce three nonlinear coefficients *h*_*e*_, *h*_1_ and *h*_2_, leading to a nonlinear Prony series model of Schapery’s type [39] with

3.33 $$\sigma^{\prime }=2\left[G_{\infty }h_{e}\epsilon^{\prime }+h_{1}\Sigma_{k}G_{k}\int_{0}^{t}\exp\left(-\frac{t-\tau }{\tau_{k}}\right)\frac{d(h_{2}\epsilon^{\prime })}{d\tau }d\tau \right].$$

Considering (3.1), (3.33) can be simplified to

3.34 $$\sigma^{\prime }=2G_{0}\left\{h_{e}\epsilon^{\prime }-\Sigma_{k}g_{k}\left[h_{e}\epsilon^{\prime }-h_{1}\int_{0}^{t}\exp\left(-\frac{t-\tau }{\tau_{k}}\right)\frac{d(h_{2}\epsilon^{\prime })}{d\tau }d\tau \right]\right\}.$$

In order to use the nonlinear material constitutive model in an FEA, (3.34) should be integrated. A nonlinear Maxwell strain at time *t*_*n*_ is defined as

3.35 $$\epsilon_{k}^{n^{\prime }}=(h_{e}\epsilon^{\prime }){|}_{{\text{t}}_{\text{n}}}-h_{1}\int_{0}^{t_{n}}\exp\left(-\frac{t_{n}-\tau }{\tau_{k}}\right)\frac{d(h_{2}\epsilon^{\prime })}{d\tau }d\tau .$$

In the following analysis, the deviatoric strain tensor at time *t* is expressed as *ϵ*′, while at time *t*+Δ*t* it is expressed as *ϵ*′+Δ*ϵ*′. A similar notation is used for other values, hence, from (3.35),

3.36 $$\epsilon_{k}^{\text{n}+1^{\prime }}=h_{e}\epsilon^{\prime }+\Delta (h_{e}\epsilon^{\prime })-(h_{1}+\Delta h_{1})\int_{0}^{t_{n+1}}\exp\left(-\frac{t_{n+1}-\tau }{\tau_{k}}\right)\frac{d(h_{2}\epsilon^{\prime })}{d\tau }d\tau .$$

Dividing the integration time period 0∼*t*_*n*+1_ into 0∼*t*_*n*_ and *t*_*n*_∼*t*_*n*+1_ in (3.36), we have

3.37 $$\epsilon_{k}^{n+1^{\prime }}=f_{1}-f_{2},$$

where

3.38 $$f_{1}=h_{e}\epsilon^{\prime }+\Delta (h_{e}\epsilon^{\prime })-(h_{1}+\Delta h_{1})\int_{0}^{t_{n}}\exp\left(-\frac{t_{n+1}-\tau }{\tau_{k}}\right)\frac{d(h_{2}\epsilon^{\prime })}{d\tau }d\tau$$

and

3.39 $$f_{2}=(h_{1}+\Delta h_{1})\int_{t_{n}}^{t_{n+1}}\exp\left(-\frac{t_{n+1}-\tau }{\tau_{k}}\right)\frac{d(h_{2}\epsilon^{\prime })}{d\tau }d\tau .$$

Considering (3.35), (3.38) can be simplified as

3.40 $$f_{1}=-(h_{1}+\Delta h_{1})\exp\left(-\frac{\Delta t}{\tau_{k}}\right)(h_{e}\epsilon^{\prime }-\epsilon_{k}^{n^{\prime }})+h_{e}\epsilon^{\prime }+\Delta (h_{e}\epsilon^{\prime }).$$

In (3.39), it is assumed the time increment Δ*t* is small, so that d(*h*_2_*ϵ*′)/d*τ* can be replaced by Δ**(*h*_2_*ϵ*′)/Δ*t*. Hence, integration of (3.39) results in

3.41 $$f_{2}=(h_{1}+\Delta h_{1})\frac{\Delta (h_{2}\epsilon^{\prime })}{\Delta t}\left[1-\exp\left(-\frac{\Delta t}{\tau_{k}}\right)\right]\tau_{k}.$$

Substituting (3.40) and (3.41) into equation (3.37), we obtain

3.42 $$\begin{array}{rl} \Delta \epsilon_{k}^{\prime } & =\left[1-(h_{1}+\Delta h_{1})\exp\left(-\frac{\Delta t}{\tau_{k}}\right)\right](h_{e}\epsilon^{\prime }-\epsilon_{k}^{n^{\prime }})\\ & \;+\Delta (h_{e}\epsilon^{\prime })-(h_{1}+\Delta h_{1})\frac{\Delta (h_{2}\epsilon^{\prime })}{\Delta t}\left[1-\exp\left(-\frac{\Delta t}{\tau_{k}}\right)\right]\tau_{k}, \end{array}$$

where $\Delta \epsilon_{k}^{\prime }=\epsilon_{k}^{n+1^{\prime }}-\epsilon_{k}^{n^{\prime }}$ is the increment of nonlinear Maxwell strain shown by (3.42). It can be seen that, when *h*_*e*_=*h*_2_=*h*_1_=1, (3.42) simplifies to (3.8), i.e. Prony series linear VE. It can be seen therefore that the integration process for Schapery-type NVE also applies to linear VE, the latter simply being a special case. Similar to the linear VE derivation in (3.9)–(3.11), it can be written for the nonlinear case that

3.43 $$\Delta \sigma^{\prime }=2G_{0}\Delta \epsilon_{E}^{\mathrm{NV}},$$

where

3.44 $$\Delta \epsilon_{E}^{\mathrm{NV}}=\Delta (h_{e}\epsilon^{\prime })-\Sigma_{k}g_{k}\Delta \epsilon_{k}^{\prime },$$

and the nonlinear Maxwell strain increment Δ*ϵ*′_*k*_ is computed from (3.42).

Regarding the stiffness matrix, it has been pointed out [38] that an analytically derived stiffness matrix is not always obtainable; however, an accurate stiffness matrix is not absolutely necessary as an approximate one is sufficient in many cases. For the NVE material constitutive model, both an approximate analytical stiffness matrix and accurate numerical stiffness matrix will be illustrated. In order to derive the analytical stiffness matrix, the variations of coefficients increments of Δ*h*_*e*_, Δ*h*_1_ and Δ*h*_2_ will be ignored for simplification. From the general relationship between stress/strain and deviatoric stress/strain, we have the following equations:

3.45 $$\delta (\Delta \sigma )=\delta (\Delta \sigma^{\prime })+\frac{1}{3}KII:\delta (\Delta \epsilon )$$

and

3.46 $$\delta (\Delta \epsilon )=\delta (\Delta \epsilon^{\prime })+\frac{1}{3}II:\delta (\Delta \epsilon ),$$

where *δ* and Δ** represent variation and increment, respectively, *K* is the material volumetric modulus and *I* is a second-order unit tensor. Considering the variations of the increments in (3.43) and (3.44), and ignoring variations in Δ*h*_1_, Δ*h*_2_Δ*h*_*e*_, we have

3.47 $$\delta (\Delta \sigma^{\prime })=2G_{0}[h_{e}\delta (\Delta \epsilon^{\prime })-\Sigma_{k}g_{k}\delta (\Delta \epsilon_{k}^{\prime }\}],$$

and from (3.42) we also have

3.48 $$\delta (\Delta \epsilon_{k}^{\prime })=\left[h_{e}-h_{1}h_{2}\frac{1-\exp(-\Delta t/\tau_{k})}{\Delta t/\tau_{k}}\right]\delta (\Delta \epsilon^{\prime }).$$

Inserting (3.48) into (3.47), we arrive at

3.49 $$\delta (\Delta \sigma^{\prime })=2G_{0}\gamma \delta (\Delta \epsilon^{\prime })$$

and

3.50 $$\gamma =\left\{h_{e}-\Sigma_{k}g_{k}\left[h_{e}-h_{1}h_{2}\frac{1-\exp(-\Delta t/\tau_{k})}{\Delta t/\tau_{k}}\right]\right\}.$$

Substituting (3.47)–(3.49) into (3.45) and (3.46) and simplifying, we can derive

3.51 $$\delta (\Delta \sigma )=\left[2G_{0}\gamma \left(\text{I}-\frac{1}{3}II\right)+\frac{1}{3}KII\right]:\delta (\Delta \epsilon ).$$

All elements of the material stiffness matrix can now be derived from (3.51). Computations have shown that the stiffness matrix in (3.51) is not only applicable for the NVE case (where at least one of the coefficients of *h*_1_, *h*_2_ and *h*_*e*_ is greater than 1), but also works well for both the VE case (where *h*_1_=*h*_2_=*h*_*e*_=1) and the case of VEVP. In case a more accurate stiffness matrix is needed, a numerical method can be used. Using Voigt notation, let

3.52 $$\left(\sigma_{1}\;\sigma_{2}\;\sigma_{3}\;\sigma_{4}\;\sigma_{5}\;\sigma_{6})=(\sigma_{11}\;\sigma_{22}\;\sigma_{33}\;\sigma_{12}\;\sigma_{13}\;\sigma_{23}\right)$$

and

3.53 $$(\varepsilon_{1}\;\varepsilon_{2}\;\varepsilon_{3}\;\varepsilon_{4}\;\varepsilon_{5}\;\varepsilon_{6})=(\gamma_{11}\;\gamma_{22}\;\gamma_{33}\;\gamma_{12}\;\gamma_{13}\;\gamma_{23}),$$

we have

3.54 $$D_{ij}=\frac{\partial \Delta \sigma_{i}}{\partial \Delta \varepsilon_{j}},$$

where *σ*_*i*_ and *ε*_*j*_ are the stress and strain components in Voigt notation, shown by (3.52) and (3.53), respectively, *γ*_*ij*_ is the strain component in Voigt format, and *D*_*ij*_ in (3.54) is the element of the material stiffness matrix. Thus, we can use (3.54) to compute the material stiffness matrix directly using a numerical method. The subroutine for the stress–strain relation is called six times for the computation of the stiffness matrix for each material point; therefore, the potential increase in accuracy comes at the cost of computational efficiency. However, (3.54) is available for the case where an analytical stiffness matrix cannot be obtained.

The final consideration in this material constitutive model is the format of the nonlinear coefficients. Poons & Ahmad [40] showed that, for material with Schapery-type nonlinearity, the nonlinear coefficients in 3D cases can be taken as

3.55 $$\begin{array}{rl} h_{1} & =1+\beta_{1}(\epsilon_{11}+\epsilon_{22}+\epsilon_{33}+\epsilon_{12}+\epsilon_{23}+\epsilon_{31}),\\ h_{2} & =1+\beta_{2}(\epsilon_{11}+\epsilon_{22}+\epsilon_{33}+\epsilon_{12}+\epsilon_{23}+\epsilon_{31})\\ \;\mathrm{and}\;h_{e} & =1+\beta_{e}(\epsilon_{11}+\epsilon_{22}+\epsilon_{33}+\epsilon_{12}+\epsilon_{23}+\epsilon_{31}), \end{array}\}$$

where *β*_1_, *β*_2_ and *β*_*e*_ are material empirical constants. Equations (3.42)–(3.44) and (3.51) or (3.54) can be applied to create a user-defined material subroutine in ABAQUS for the FEA of materials with a simplified form of Schapery-type nonlinearity.

### (d) Nonlinear viscoelastic–viscoplastic material constitutive model

Consideration of NVE requires the introduction of nonlinear coefficients, as shown in (3.34), to increase or decrease the viscous component relative to the elastic part in the deformation, leading to better fitting between test and FE modelling. However, modelling experience has shown that nonlinear coefficients alone cannot meet this requirement as large nonlinear coefficients result in convergence problems. In this case, it may be necessary to also consider VP deformation. This may occur if the NVE stress is sufficiently high to actuate VP flow. This condition can be expressed as: if the NVE equivalent stress *σ*_*e*_ is higher than the current yield stress *σ*_*h*_, VP flow is actuated. It is also assumed that, when VP flow occurs, the increment of current deviatoric strain tensor Δ*ϵ*′ during Δ*t* is divided into two parts, a nonlinear NVE strain increment contribution and the VP strain increment Δ*ϵ*_*p*_ part. The NVE strain increment, from (3.42) to (3.44), can be expressed as a function of the deviatoric strain increment Δ*ϵ*′, i.e.

3.56 $$\Delta \epsilon_{E}^{\mathrm{NV}}=\Omega (\Delta \epsilon^{\prime }),$$

in a case without VP flow being actuated for the given nonlinear coefficients and Maxwell strains. In an NVEVP case, similar to (3.19), the NVE strain increment can be written as

3.57 $$\Delta \epsilon_{E}^{\mathrm{NV}}=\Omega (\Delta \epsilon^{\prime }-\Delta \epsilon_{p}).$$

Using a calculation procedure similar to that between (3.23) and (3.30), it can be derived that

3.58 $$\Delta \epsilon_{E}^{\mathrm{NV}}=\Omega (\Delta \epsilon^{\prime })-\Delta \epsilon_{p}\hat{1},$$

where for an NVEVP case

3.59 $$\hat{1}=h_{e}-\Sigma_{k}g_{k}\left[h_{e}-(h_{1}+\Delta h_{1})h_{2}\frac{\tau_{k}}{\Delta t}(1-e^{-\Delta t/\tau_{k}})\right].$$

It can be shown that, when *h*_1_=*h*_2_=*h*_*e*_=1, (3.59) simplifies to (3.25) for the linear VE case. Similarly, the direction of Δ*ϵ*_*p*_ can be shown to be

3.60 $$\text{n}=\frac{3}{2}\frac{\sigma^{\prime }}{\sigma_{e}}\equiv \frac{3}{2}\frac{\sigma^{{\mathrm{tr}}^{\prime }}}{\sigma_{e}^{\mathrm{tr}}},$$

where ***σ***^tr′^ is a trial NVE deviatoric stress tensor computed from equation (3.57) by ignoring the possible VP strain Δ*ϵ*_*p*_. The nonlinear algebra equation to compute the equivalent VP strain increment is

3.61 $$\Delta p-\phi (\sigma_{e}^{\mathrm{tr}}-3G_{0}\Delta p\hat{1}-\sigma_{h})\Delta t=0,$$

where $\sigma_{e}^{\mathrm{tr}}$ is computed according to the NVE material constitutive model ignoring the possible VP strain and $\hat{1}$ is calculated from (3.59). As for the required stiffness matrix, (3.51) can be applied, as discussed earlier. Equation (3.61) is similar to equation (3.31); however, $\sigma_{e}^{\mathrm{tr}}$ should be computed according to Schepary’s type of NVE, shown by equation (3.34).

## 4. Results and discussion

### (a) Validation of material models and inverse finite-element method

**Figure 4.**
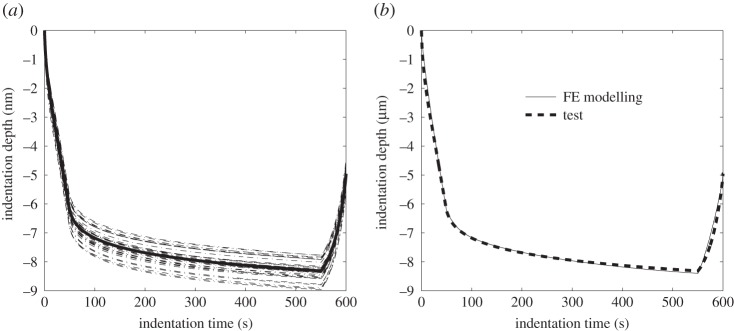
(*a*) Indentation depth–time curves for UHMWPE (average in bold); (*b*) the fitting between FE modelling and test by inverse FE technique using the VEVP material constitutive model.

In order to validate the numerical method, an ultra-high molecular weight polyethylene (UHMWPE), which had previously been characterized, was tested. This material is known to show viscoelastoplastic characteristics and the validation uses the VEVP material model. The UHMWPE preparation has been reported in [41], and the method described in §2a was used to make an indentation sample. A nanoindentation test comprising a 6×6 array of indents, with a step length of 100 μm in both *x*- and *y*-directions, was conducted on the central region of the sample. The depth–time curves are shown in figure 4*a* and the average (seen as the bold solid line in the figure) was then used as a reference curve for the inverse FE modelling. Using the proposed VEVP material model and IFEM technique, the properties of the UHMWPE were determined, as shown in table 1. It can be seen in figure 4*b* that these parameters enable an excellent fit between the experimental load–depth curve and that predicted with the FE mode, thus confirming the applicability of the FEA indentation model, the VEVP material model and the IFEM technique for finding best-fit material parameters.

**Table 1.** Material property parameters of UHMWPE determined from indentation testing and inverse FE modelling using a VEVP material model.

material model	VEVP
*E*_*in*_ (MPa)	666.2268
*g*_1_	0.4012
*g*_2_	0.1193
*g*_3_	0.2444
*τ*_1_ (s)	0.2833
*τ*_2_ (s)	214.8038
*τ*_3_ (s)	13.4790
*σ*_*s*_ (MPa)	19.1032
*m*	2.9500
*η*(μm^2^ N^−1^)	0.2038
*ζ* (s^−1^)	0.2038

**Figure 5.**
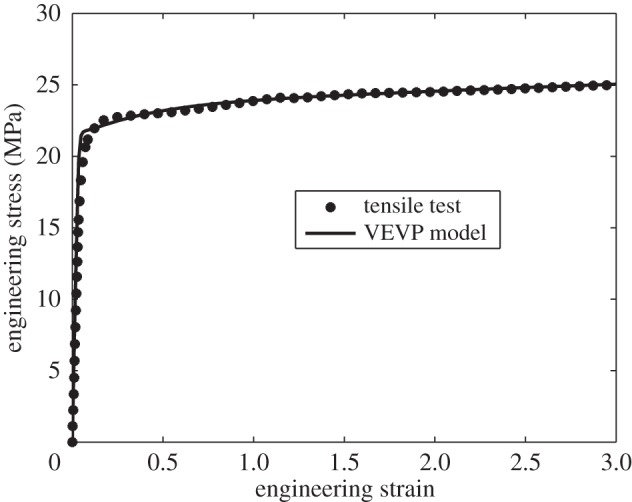
A comparison of experimental and predicted tensile test results performed in order to validate the VEVP material model and IFEM based on nanoindentation.

For further validation, the results from a uniaxial tensile test at a constant engineering strain rate of 0.2 s^−1^ at room temperature given in [41] were predicted. A 3D FE-based model of the tensile test was performed, using the VEVP constitutive model with the material parameters given in table 1. Figure 5 shows a comparison of the experimental results from [41] and the results of the model. This shows excellent agreement, suggesting that the proposed method is able to capture the material behaviour well.

### (b) Nanoindentation of three-dimensional printed sample

**Figure 6.**
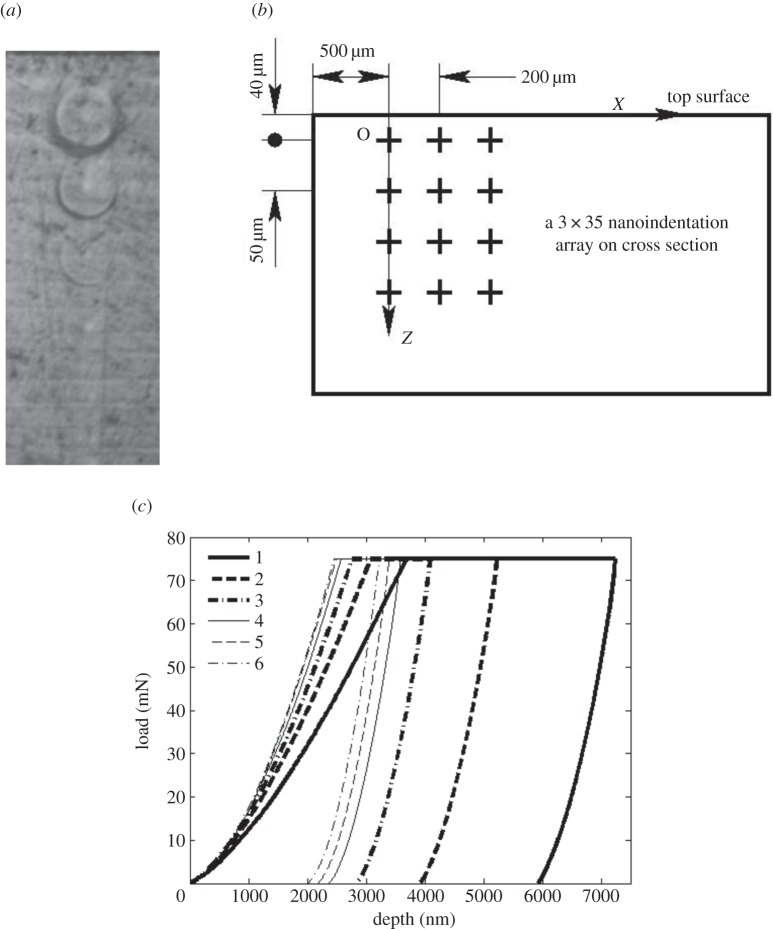
(*a*) Optical image of imprints left on the sample test surface following indentation with 75 mN loads. The distance from the top surface to the first indentation is 40 μm and the separation of subsequent indentations is 50 μm. (*b*) Schematic diagram of the 3×35 nanoindentation array on the cross section of the sample. (*c*) Typical indentation load versus depth curves with maximum load of 75 mN, where ‘1’ is the indentation closest to the surface, each subsequent number indicating a further 50 μm below the surface.

Having validated the proposed method, it was then applied in the determination of the material properties of a 3DP sample. The sample was prepared using the method described in §2a and a 3×35 array of indentations was performed to determine the spatially resolved material properties. In each column, the load was held constant, but across the columns the load was varied with 25, 50 and 75 mN maximum loads used, respectively. In the *z*-direction the depth interval was 50 μm, with the first indentation at a depth of 40 μm from the sample surface. The distance to the side edges was more than 500 μm. The matrix of points is illustrated in figure 6*b*. The procedure for the indentation was described in §2b.

Figure 6*a* shows the indentation marks left on the test surface during one of the experiments, using a 75 mN indentation load. It can be seen that the residual imprint of the indentation varies with the *z*-position, suggesting, at least qualitatively, that there are material variations in the *z*-direction. As the largest residual impression is closest to the top surface, this would indicate that hardness increases with depth below the surface and this is confirmed in figure 6*c*. The greatest depth is seen with the indentation closest to the top surface (labelled 1), and then the maximum depth is gradually smaller for subsequent indentations (labelled 2, 3, … and 6, respectively). After around eight indentations, the penetration depth becomes independent of *z*. The figure shows the load–depth curves for the first six indentations, each an additional 50 μm in the *z*-direction.

**Figure 7.**
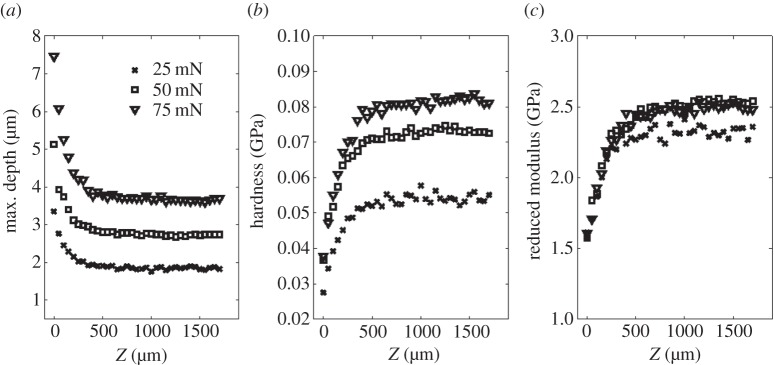
Typical nanoindentation result on VeroClear cross section for a 3×35 indentation array with a 50 μm radius spherical indenter. The variations in (*a*) maximum depth, (*b*) hardness and (*c*) reduced modulus, with respect to depth in the *z*-direction, are shown for maximum loads of 25, 50 and 75 mN.

Figure 7 shows the variation of penetration depth, hardness and reduced modulus with increasing *z*, i.e. distance below the top surface, while keeping *x* constant. It can be seen that the maximum achieved indentation depth is closest to the top surface (figures 6*c* and 7*a*). As the indentation location is increased in the *z*-direction, the maximum indentation depth decreases, appearing to reach an asymptotic value at around 500 μm. The hardness and reduced modulus show similar behaviour. It is noted also that the hardness is indentation load dependent, as discussed in [42], while the reduced modulus is relatively load independent if the indentation load is at least 50 mN. These results correlate well with the qualitative observation of varying residual imprints, indicating that the printed VeroClear material properties are *z*-dependent, and, in particular, depend on the number of printed layers of material. Because the mechanical property of this reactive inkjetted material should only depend on the extent of the cross-linking during post-build UV illumination, it is supposed that the primary cause of this phenomenon is the varying number of exposures to UV, i.e. the bottom-most layer will have the same number of exposures as there are layers of material and is more likely to have saturated the cross-linking opportunities because of the transparency of the printed material, while the top layer will have been subjected to only one UV exposure.

### (c) Inverse finite-element analysis results

**Figure 8.**
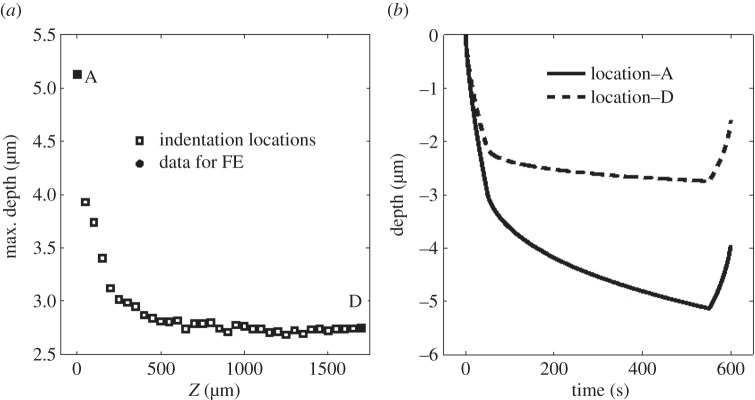
(*a*) The maximum depth as a function of *z*-position with 50 mN indentation load, selected points labelled ‘A’ and ‘D’. (*b*) Depth versus time curves for positions A and D used in inverse FE modelling.

The IFEM described in §2d was then performed at two locations, enabling assessment of the technique when there are spatially varying properties, such as the variation with depth seen in the previous section. The two locations were at *z*=40 and 1740 μm, representing a location near to the surface where the properties are changing rapidly, and within the bulk where the material properties are largely independent of *z*. A maximum load of 50 mN was used. The maximum depth versus *z*-position at this load is shown in figure 8*a* with the selected ‘surface’ and ‘interior’ positions labelled ‘A’ and ‘D’, respectively. The depth–time curves at these positions are shown in figure 8*b*.

**Figure 9.**
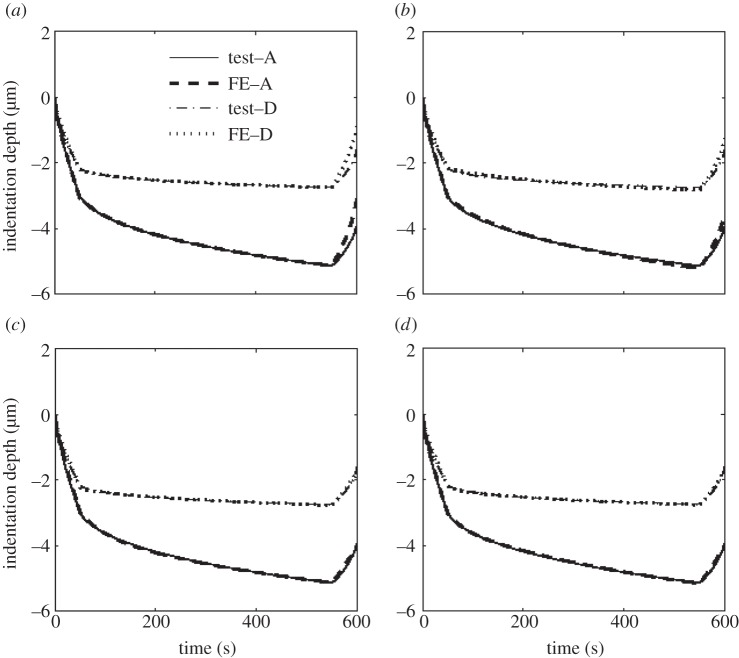
Typical fitting of depth versus time between the nanoindentation test and FE modelling with (*a*) linear Prony series (VE), (*b*) NVE constitutive model, (*c*) VEVP constitutive model and (*d*) NVEVP constitutive model.

From the nanoindentation results, an assessment of the applicability of each of the proposed constitutive models was made, by taking the depth versus time plots and using them as an input to the objective function (equation (2.1)). The IFEM was then employed for each to determine the optimum parameters of the constitutive models that would best reproduce the experimental results. The comparisons between experiment and numerically obtained depth versus time plots are shown in figure 9 for each constitutive model.

It is can be seen in figure 9*a* that, for the VE material model, both the loading stage and load-holding stage can be fitted well; however, the FE predicted elastic recovery during the unloading stage is much higher than that seen experimentally. The introduction of nonlinear coefficients by using the NVE model improved this, as seen in figure 9*b*. Mechanistically, the aim of introducing the nonlinear coefficients, *h*_1_, *h*_2_ and *h*_*e*_, in equation (3.34) is to increase the viscous strain component in the total strain for the given loading conditions, thus to increase the viscous deformation which is unrecoverable during the unloading stage, leading to better fitting. However, computational experiments showed that the nonlinear coefficients could not be made sufficiently high to enable the FE predicted elastic recovery fit exactly to the experimental curve. This is because too high nonlinear coefficients result in failure of the modelling procedure due to convergence problems. The optimal results using the NVE material model, therefore, lie between the VE and VEVP material models. Owing to the aforementioned limitation of the nonlinear material model, the VEVP and NVEVP material models were thus introduced, with the aim that the over-predicted FE elastic recovery can be suppressed by VP deformation during the loading and holding stages. Figure 9*c*,*d* illustrates that both these models are significantly more capable of modelling the unloading curve than the VE models. The relative merits of these models are difficult to assess just by visual assessment of the curve fits in figure 9; therefore, a quantitative method of comparison was developed.

To quantify the ability of a material model to capture the behaviour during indentation a relative residual *R*_r_ was computed. This is given by

4.1 $$R_{r}=\sqrt{\frac{1}{n}\Sigma \left[\frac{{\left(S_{Fk}-S_{Tk}\right)}^{2}}{abs(\min(S_{Tk}))}\right]}\times 100,$$

where *S*_*Fk*_ and *S*_*Tk*_ are the depth at time *t*_*k*_ by FE modelling and experimentation, respectively, *n* is the division number during the test (or FE modelling). *R*_r_ represents the relative difference of indentation depth between test and FE modelling, i.e. the closer *R*_r_ is to zero, the closer the agreement between the FE model and the experiment. The results are given in table 2, which shows that the NVEVP model fits the experiment the closest, suggesting that this constitutive model is the most appropriate to characterize the mechanical response of the VeroClear to complex loading.

**Table 2.** Relative depth residuals of different locations with different material models.

location	A				D			
material model	VE	NVE	VEVP	NVEVP	VE	NVE	VEVP	NVEVP
residual (%)	5.2554	3.1868	1.7295	1.0554	6.8946	4.1823	1.7505	1.7086

**Figure 10.**
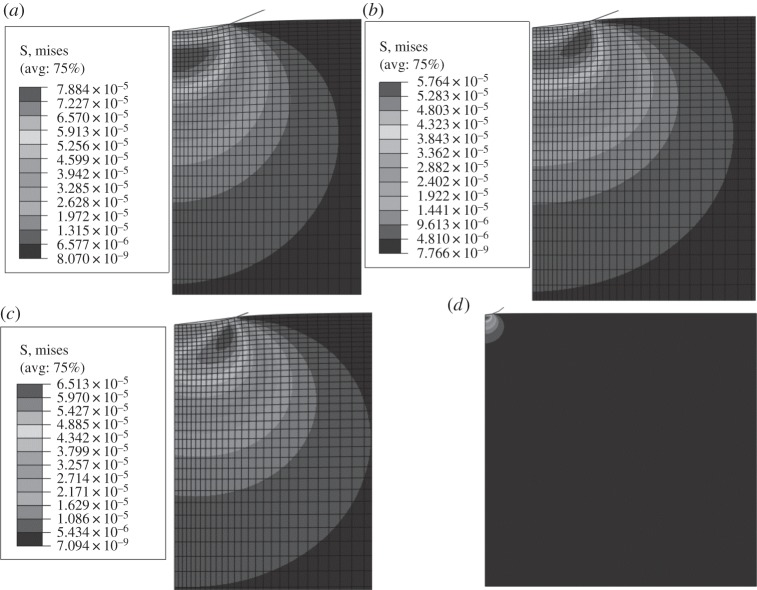
Von Mises stress distributions at immediate maximum indentation load for (*a*) VE, (*b*) VEVP and (*c*) NVEVP material constitutive models for location A (figure 8) with an indentation load of 50 mN. Note that the displayed stress unit in the diagrams is N μm^−2^. (*d*) The full model view for the VE model, showing that the geometry of the model is sufficiently large for the given material and load.

In order to determine the root cause of the variance in fits seen in table 2, the predicted stress distributions at attaining maximum load in the FE model for the various material models were compared (figure 10). The differences in stress distribution are striking. When using a linear Prony series, a high stress field is located immediately below the indenter (figure 10*a*). With the VEVP model, the high stress field is near to the contact edge area, where VP flow occurs. Subsequently, this high stress region moves with the edge when the indentation load increases (figure 10*b*) and during the load hold stage. Similarly, for the NVEVP model, the high stress field is located near the contact edge, and moves towards and with the contact edge when the indentation load increases (figure 10*c*). With such varying and complex stress distributions, differences in the closeness of the fit of experimental and predicted load–depth plots should be expected. The total model image of the FE model used for figure 10*a* is shown in figure 10*d*. As can be seen, the stress-influenced area during indentation, compared with the total model, is rather small; the stress-affected area is far away from any model edge except the top surface. This suggests that the model sizes shown in figure 1*b* are sufficiently large to be unaffected by the geometry and boundary condition simplifications used in the model.

The evaluated material parameters using the various material constitutive models for indentation locations A and D are shown in table 3, where *E*_*in*_ is instantaneous modulus, *τ*_1_, *τ*_2_ and *τ*_3_ are relaxation times and *g*_1_, *g*_2_ and *g*_3_ are the corresponding relative relaxation moduli (see equation (3.3)), *σ*_*s*_ is the initial hardening yielding stress for least depth residual (see equation (3.18)), *β*_*e*_, *β*_1_ and *β*_2_ are the nonlinear coefficients of the NVE and NVEVP material constitutive models (see equations (3.34) and (3.55)) and *ζ* and *η* are VP parameters, see (3.16) and (3.17).

**Table 3.** Evaluated material properties by inverse FE technique.

location	A				D			
material model	VE	NVE	VEVP	NVEVP	VE	NVE	VEVP	NVEVP
*E*_*in*_ (GPa)	1.7180	1.7568	1.2958	1.2273	2.5112	2.1354	1.9428	1.8959
*τ*_1_ (s)	0.2527	0.2734	0.3809	0.2780	0.05000	0.06677	0.3336	0.5277
*τ*_2_ (s)	267.2363	265.5842	263.8945	230.9193	270.4045	270.0201	261.2826	301.0981
*τ*_3_ (s)	35.5710	35.3632	48.8380	33.6846	34.6057	34.0225	48.9370	20.2626
*g*_1_	0.4630	0.4875	0.2364	0.2846	0.4676	0.4427	0.1545	0.1950
*g*_2_	0.1778	0.1949	0.3511	0.3712	0.1283	0.2169	0.2837	0.2380
*g*_3_	0.2144	0.2139	0.1769	0.1817	0.08491	0.08537	0.05381	0.2025
*σ*_*s*_ (MPa)	—	—	21.3424	29.5426	—	—	40.1703	51.5038
*β*_*e*_	—	4.9777	—	15.0102	—	21.4997	—	21.9566
*β*_1_	—	0.0000	—	0.0000	—	0.0000	—	0.0000
*β*_2_	—	2.6936	—	7.7964	—	11.8238	—	11.1791
*ζ* (*s*^−1^)	—	41.1684	6.4944	7.0912	—	33.5365	22.1888	33.4143
*η* (μm^2^ N^−1^)	—	41.1684	6.4944	7.0912	—	33.5365	22.1888	33.4143

The results in table 3 show that the highest instantaneous modulus is predicted by the VE material model while, for the VEVP and NVEVP material models, similar lower moduli are predicted. With a small VP strain rate, $\dot{p}$, in (3.17) for the given flow function as shown by (3.16), the effects of both *ζ* and *η* to the stress difference of $\sigma_{e}-\sigma_{h}=1/\eta \sin h^{-1}(\dot{p}/\zeta )\approx \dot{p}/\eta \zeta$ are nearly equal, although *ζ* and *η* have different physical meaning and dimensions. In order to have higher computational efficiency, it was assumed that *ζ* and *η* have the same mathematical value with different dimensions, thus a variable can be reduced in the inverse FE modelling (note that this is acceptable for the case of small deformation and low strain rate but cannot be generally assumed). This can be seen in table 3, which also shows that VP parameters in the NVEVP model are higher than in the VEVP model, especially at location D. This is because the use of nonlinear coefficients in the NVEVP model results in a higher viscous deformation, hence, less VP deformation is required to fit to the experimental data. As a result, the corresponding initial yield stresses are also higher for the NVEVP model. However, generally the indentation depth is not sensitive to these VP parameters for the Veroclear material. This is the why discrete values are obtained in the table.

Although the instantaneous moduli obtained for the VEVP and NVEVP models are nearly the same, as shown in table 3, the relative relaxation moduli and the relaxation times are somewhat different. This is because of the addition of the nonlinear coefficients in the NVEVP model leading to higher viscous deformation. The computations have shown that for normal simulations, the coefficient of *h*_1_ (see (3.33)) is restricted to nearly *h*_1_≈1 or *β*_1_≈0; *β*_1_ should be less than the order of 10^−4^ to maintain model convergence and best fitting. In order to simplify the inverse FE modelling, it was assumed that *β*_1_=0 for best fitting. This is also shown in the table.

The results show that, regardless of the constitutive model, the instantaneous elastic modulus is predicted to within 20% and is the dominant contribution to the material behaviour. Each model captures a different aspect of the deformation, with detailed differences at the level of the individual parameters. However, the parameters are largely of the same order of magnitude, suggesting that the primary physical mechanisms are being captured by each model in a similar way, particularly when the VE and VEVP models are compared. The differences in the models are larger at greater *z*-depths, suggesting that the material that has suffered more UV illumination develops a greater material nonlinearity, potentially reflecting a change in micro- and molecular structure. Within each model, it can be seen that the evaluated instantaneous modulus within the body of the sample is approximately 50% greater than that at the top surface. This evidence once again suggests that 3D inkjet printing can result in significant variation in material properties throughout a part, immediately post manufacture. If controlled, this could lead to the control of part properties through a volume and raises the potential for an extra design freedom for engineering component manufacture not available to traditional manufacturing techniques. It should be noted, however, that the properties of the material, and their spatial variation, may change with time as the material reacts to environmental conditions. The method described in this paper will be used to investigate this in future work.

## 5. Conclusion

An inverse analysis technique has been developed that combines nanoindentation with FE modelling. The primary purpose for developing this technique was to enable measurement of material property variations in 3D printing components, and a range of potentially relevant material models were developed and tested. Two characteristic locations in a 3DP sample were examined using the proposed method, near to the surface, where nanoindentation results suggest large spatial variations exist, and within the bulk, where the properties appear to be independent of location. In both cases, the method was able to determine the material properties with small residual errors between the model predictions and the nanoindentation-measured depth–time curves.

It can be concluded, therefore, that the proposed method can accurately determine spatially resolved properties as long as the constitutive material model used in the analysis is capable of fully representing the material behaviour in the test. It can be further concluded that the method can be used to investigate manufacturing processes which induce spatial variation of properties and that the accurate, spatially resolved material parameters thus derived can also be used in models to predict the performance of the manufactured part in-service.

## Data accessibility

All data are stored centrally at the University of Nottingham. Contact Ian Ashcroft at ian.ashcroft@nottingham.ac.uk for access details.
